# Capitalizing upon the Attractive and Addictive Properties of Massively Multiplayer Online Role-Playing Games to Promote Wellbeing

**DOI:** 10.3389/fpsyt.2016.00167

**Published:** 2016-10-17

**Authors:** Gabriel Thorens, Joel Billieux, Pierre Megevand, Daniele Zullino, Stéphane Rothen, Sophia Achab, Yasser Khazaal

**Affiliations:** ^1^Addictology Division, Mental Health and Psychiatry Department, Geneva University Hospitals, Geneva, Switzerland; ^2^Laboratory for Experimental Psychopathology, Psychological Sciences Research Institute, Catholic University of Louvain, Louvain-La-Neuve, Belgium; ^3^Neurology Division, Clinical Neuroscience Department, Geneva University Hospitals, Geneva, Switzerland

**Keywords:** game, serious game, gamification, health, video games, MMORPG

## Introduction

Forty years after they were first introduced to the general public, video games have become firmly established into the mainstream of entertainment media. In parallel, early adopters and enthusiasts have become active scientists. As a result, interest in video gaming practices has shifted from an exclusive niche leisure activity to extensive social, commercial, cultural, medical, and psychiatric issues. An increasing number of studies on game-based treatments for various mental health disorders suggest that these approaches have the clear potential to promote cognitive and behavioral changes as well as symptom relief (1–10).

Game-based approaches for mental health disorders are still in an early stage of development and validation. Some interesting results have emerged, however. The inclusion of gaming tools within digital therapies has been assessed in several trials that studied participants with various sociodemographical profiles and disorders (11–13), including promising preliminary work in participants suffering from psychotic disorders (8, 12). Controlled studies on the assessment of digital serious games targeting depression (2, 11) and cognitive training in older adults (4) found promising findings, as did controlled studies on non-digital serious games developed to reduce delusional convictions in psychotic patients (1) or to promote smoking cessation (5). Also, two randomized, controlled studies showed that playing casual video games 30 min per day during 1 month (*Tetris, Bejeweled*) had positive effects on depressive and anxious symptoms (14–16).

It was furthermore shown (in spite of controversy) that game and video game practice may have a positive impact through the transfer of cognitive skills acquired in the game to functional competences (17, 18), although see Ref. (19, 20). A meta-analysis (21) on the effect of video game play on information processing showed minor effects on executive functions and small to moderate effects on visual, auditory, spatial imagery, and motor skills.

Another meta-analysis on the effect of brain-training games in older adults (22) showed a significant, if very small, improvement in cognitive testing after training, suggesting that some transfer of competence had occurred (albeit in a limited fashion).

Data on naturalistic use (in milieu use outside the scope of specific research programs) of internet-based therapies suggest that these programs are appealing for many users, particularly so for people who could benefit significantly from such treatments (23). Naturalistic use, however, appears associated with high levels of attrition. For instance, in a study of an internet treatment program for depression, less than 4% of community users completed at least three modules out of five compared with 53.8% of participants in the controlled trial (24–26). Attrition issues were also observed across digital games-related studies. Less than one-third of the 1622 adolescents allocated to a computerized game for binge drinking prevention returned to the second session and none of them completed all five sessions (27).

One of the ways to tackle the problem of attrition would be to develop interventions that succeed in increasing the commitment of the participants, possibly *via* the use of serious games tools (28). The important retention problems encountered in internet treatments, particularly among spontaneous community users, contrasts with the great success of games played for leisure, and, especially, massively multiplayer online role-playing games (MMORPGs) (29), which are sometimes associated with addictive use (30, 31). This opinion paper discusses how the attractive properties of video games, particularly those of MMORPGs, given their success and online setting, could be mobilized to promote wellbeing.

## Specific Potentially Attractive and Addictive Components in Video Games and Their Potential Use for Wellbeing

Considering the attractive properties of video games as a single entity is reductive. There are indeed multiple types of video games, and an exhaustive listing of game types and their specific attractive properties is beyond the scope of this article. We will focus on attractive components common to all video games, with a specific focus on MMORPGs, since they have specific structural characteristics that could be useful for developing health-oriented video games. In the next sections, we will provide a (not necessarily exhaustive) list of video game components that have often been related to addictive use in past research (32), but which will be discussed here in terms of their potential to optimize interventions that promote wellbeing.

## Reward Conditioning Scheme

Video games offer countless opportunities to optimize the distribution of reinforcers. The pioneering work of Schultz and colleagues on rewards (33) has shown that the rate of reward distribution may condition the intensity of dopamine release in the ventral tegmental area (a group of neurons in the midbrain that is a critical component of the reward system) and that the expectation of a reward can be as incentive as the reward itself. It is worth emphasizing that in commercial video games, in contrast to “real life” (one can strive all one’s life for an ultimately unattainable goal), rewards are generally distributed in an optimized manner. Most video games are conceptualized to reward players in a fair manner, and the reward progression is harmonious and relatively predictable. When a player starts a game, the challenges are easy and the progression is fast. Thus, almost all MMORPGs are designed so that the time and effort required to “level up” (a major in-game reward whereby the player’s avatar improves their abilities) increase progressively (i.e., 5 min of play are necessary to progress from level 1 to level 2, whereas 3 h are necessary to improve from level 60 to level 61) (34). In real life, many of the skills that people have or desire to acquire follow the opposite rule; for instance, learning to play the violin is unrewarding during the first few years and becomes easier and more enjoyable the more one’s skill improves. Furthermore, in MMORPGs, the rate of progression is always in a positive direction: the avatar’s strength and power grow steadily during the game, and developers do not usually integrate avatars that age or develop chronic diseases or cognitive decline.

In the next sections, we provide illustrations of how MMORPG-based reward distribution could be used in games designed to increase wellbeing. In the case of video games that aim to improve physical condition (exergaming, for example a video game coupled with a running treadmill), a solution to compensate for the frustration of the difficult first steps of practicing (as in the violin example) would be to increase the amount of rewards at the beginning of the game. Users could initially gain in-game rewards and levels after running for short distances and, therefore, experience the subjective impression that the task is rapidly rewarding. Such a view is supported by a recent review on exergaming (35).

A more tentative proposition would be a game designed to overcome social phobia, which could take the form of a multiplayer game where players are encouraged to have and rewarded for having face-to-face video conferences. In such a game, every social contact should be rewarded according to the reinforcement schedule depicted earlier, so that the participant fully appreciates his or her progression. Using such a game is susceptible to optimize the learning process of social exposure, in comparison to real-life situations where no direct positive feedback follows an exposure exercise (the shopkeeper will rarely congratulate the patient for sustaining their gaze and speaking clearly).

These rewards mechanisms can also have downfalls. The loss of the novelty effect is one: the generalization of the so-called gamification of everyday activities tends to reduce the effect of virtual rewards, thus the incentives to receive virtual rewards could progressively decrease. It is also important to bear in mind that in-game rewards remain intangible; strategies to increase behavioral modification could link virtual rewards to real ones to increase their attractiveness (for instance, winning a real pair of running shoes could be linked to an in-game achievement). Similar strategies have been applied for a long time in contingency management for the treatment of substance use disorders with positive outcomes overall (36).

Rewards should be conceptualized as a means to promote wellbeing and not as an end in themselves to avoid the development of an addiction to the game mechanisms. Take the case of *Zombies, Run!*, an audio/video game that couples running exercises to missions where the player must escape zombies chasing them. Running with headphones, the player hears where zombies are lurking and how to escape them. The more they run effectively, the more they gain levels and bonuses. A problematic use of this game could appear if the player’s purpose becomes not to enhance their running ability, but rather to progress in the game as fast as possible. As they become addicted to the in-game rewards, they will start to play the game while driving their motorcycle or car to be more effective. This supposition might sound unrealistic, but it is in fact the core mechanism of the so-called “free-to-play” games, where the player has the choice to either play a game for free, but must play for very long to get a reward, or pay to progress much faster and avoid the long wait (37).

## Never-Ending Games

Massively multiplayer online role-playing games, as opposed to single-player role-playing games, are endless. The developers constantly update the game to implement new features and provide every player new tasks to accomplish, often on a daily or weekly basis. The game will stop only when its popularity decreases and the developers stop maintaining the dedicated servers. Even then, sometimes, private communities continue to maintain the game on their own and provide continuity in the game environment for the most committed players.

It is obviously difficult to provide the same continuity in so-called serious games, whose development and validation are generally tied to studies that themselves depend on grants with a fixed and limited amount of time and money. Commercial video games are extensively tested before they are eventually released. After release, constant updates and improvements have to be implemented. Players directly contribute to these ameliorations through constant feedback on their gaming experience.

Open-source games for health could represent a potential solution (38). In open-source software, the computer code of the software is available for anyone to modify; thus, users could modify themselves the game’s content and continue the development and maintenance of validated games, i.e., games whose potential for health improvement was demonstrated in controlled trials. The risk with open-source software, however, is that games could evolve in an undesirable direction. If any player is free to modify or implement new game features, ill-intentioned players or hackers could intentionally introduce inappropriate content or threaten other players (cyberbullying).

## Social Interaction Between Players

The more a game is popular, the more players will be attracted. In 2009, the MMORPG *World of Warcraft* reached more than 12 million players worldwide. This high number of players obviously offers the possibility for everyone to find affinities with other players and progress together. Game developers are aware of the key role of social interactions as a factor for the success of a video game (39), and MMORPGs are thus designed to allow people to regroup in social virtual networks (often called “guilds”) or to communicate in real time while playing. If the same principle is applied to a game that addresses social phobia, the large amount of people playing this game will help to alleviate the stigma of the diagnosis and make the players feel less alone and better understood regarding their specific difficulties. Furthermore, the peer reinforcing effect would be beneficial for users.

Another example of peer reinforcing effect is a Swiss initiative proposing a self-help group on Facebook for tobacco addiction. The objective was to set a common date for participants to quit smoking; the members of the group would then help each other to keep motivated.^1^ This initiative was more successful than expected, and developers of games for health could be inspired by this type of experience.

One of the negative counterparts of the social aspect of games is the risk of recreating within the game communities that are stigmatized or excluded in real life. Clinical practice shows that people with social interaction problems also tend to have difficulties in virtual life and could reproduce their difficulties and poor coping strategies in games (40). Video game companies have already had to deal with antisocial in-game behaviors. Their employees must constantly monitor the interactions between players and sanction or ban problematic players. It would, therefore, be fundamental to avoid cyberbullying by potentially antisocial individuals (41).

## Appealing Content

Developing a video game requires a large multidisciplinary team of programmers, artists, scenarists, 3D motion specialists, etc. Obviously, a task generated with the tools traditionally used in experimental psychology and psychiatry (software such as *E-Prime* or *Psychopy*) will not have the same visual or emotional impact as software developed by such a multidisciplinary professional team. As an illustration, a video game to treat anxiety *via* biofeedback, called *Mindlight*,^2^ was developed in collaboration with a video game company to combine the video game industry’s *savoir faire* with scientific knowledge. The result is designed to propose the appealing visuals of a state-of-the-art video game with a scientific rationale.

## Competition

Along with social interaction, competition is a key component in the attractiveness of MMORPGs and of video games more generally (42). The net democracy or “equal chances for all” is an important incentive and a rewarding possibility. Someone who is 1.5 m tall (4 ft 11″) has very low chances of becoming an NBA basketball star, no matter their skill. Video games offer a chance for everyone to start with the same characteristics; as such, the balance between avatars is a main concern for game developers. Competition is described as an important motivation for MMORPG players (43), and most games are designed to match individuals with similar skill levels together. Thus, the impression of having a fair competition enhances the motivation to perform a task. In exergaming for example, the level of the player’s performances could be monitored, and the possibility of competing against virtual avatars or other individuals constantly matched to their level is expected to enhance their motivation to play. Thus, if someone aged 33 has the running capacity of a 70-year-old, they will not be able to compete with runners their age, but will likely not be interested or allowed to join the senior category in a traditional running competition. If, on the other hand, the same runners are represented by their avatar performance without any mention of age, they could have the impression to progress and achieve good performances in their own avatar category.

Competition could also become counterproductive in wellbeing-oriented games, and, once again, if the end goal becomes the competition by itself, it could divert the game from its original objective and lead to negative outcomes.

## Games are Virtual

Video games give opportunities to experience sensations otherwise too dangerous, forbidden or inaccessible. *The virtual-reality headset technologies (i.e., the “Oculus Rift” system purchased by Facebook) become affordable and broadly available and could greatly enhance immersion*. For example, people who are afraid of driving or flying could play realistic simulations to have a first experience, and eventually gain knowledge and confidence in these activities (44). This could then lead to a better motivation to try these activities in real life. As described before, the generally established efficacy of virtual-reality treatments of anxiety disorders (45) (e.g., exposing individuals to virtual spiders for arachnophobia) sustains this argument. The main advantage of this approach is to provide a realistic but safe environment, free from any real threat, that makes the patients more inclined to accept the treatment (there are no real spiders during the treatment or, in the case of acrophobia, no real cliffs), but that still triggers real manifestations of fear. On the opposite, it could be argued that because there are no real consequences to a virtual action, dangerous behaviors devoid of any concrete negative consequence could be promoted and applied to the real life, as in the example of reckless driving in video games (there are no real consequences to a virtual car crash) (46). *The use of virtual avatars could also be helpful in psychiatric treatments, as, for example, evidenced by a recent study that demonstrated how using avatar-based therapy is useful to decrease persecutory auditory hallucinations* (47).

## Conclusion

The popularity of video games has been established in the past four decades and is still growing. Nowadays, successful video games are designed for entertainment, but as researchers are analyzing the mechanisms of this success, they are more and more confident on the possibilities of designing video games for wellbeing. In this article, we formulated ideas to suggest how to take advantage of the core attractive mechanisms of MMORPGs, such as conditioning reward schemes enhanced by players’ social interactions (Table 1).

**Table 1:** **Summary of specific potentially attractive components in MMORPGs and their potential use for wellbeing**.

	Potential positive effects	Potential negative effects
Reward conditioning scheme	– Enhanced learning curve	–Reward rate optimized
	– Risk of addictive use	–Repetitive and boring tasks
Never-ending games	– Permanently available	–Constant improvement
	– Risk of addictive use	–Expensive to maintain
Social interactions	– Peer support	–Positive feedback
	–Belonging to a community – Risk of cyberbullying	–Risk of stigmatization
Appealing content	– Enhance motivation to play	– Expensive and difficult to develop
Competition	– Enhance motivation to play	–Adapted levels for all players
	– Risk of addictive use	–Risk of aggressive/antisocial behaviors
Virtual aspect of games	– Safe environments to experiment stressful situation	–Virtual environments highly adaptable
	– Can promote maladaptive or unhealthy behaviors	–Not strictly comparable to the “real life”

We also pointed out some obstacles to the realization of video games for wellbeing, such as the lack of academic resources compared to the game industry and the risk of encouraging problematic or addictive video game use. Technological progress in the field is rapidly evolving, and virtual or augmented reality will soon provide new innovative tools to further the development of video games for wellbeing.

## Author Contributions

All authors listed have made substantial, direct, and intellectual contribution to the work and approved it for publication.

## Conflict of Interest Statement

The authors declare that the research was conducted in the absence of any commercial or financial relationships that could be construed as a potential conflict of interest.

## Footnotes

^1^https://www.facebook.com/j.arrete.de.fumer.ch

^2^http://www.gainplaystudio.com/mindlight/
